# A case of methotrexate‐associated Epstein‐Barr virus‐positive mucocutaneous ulcer

**DOI:** 10.1002/ski2.108

**Published:** 2022-03-17

**Authors:** Keiko Sakamoto, Takeshi Baba, Hiroaki Takatori, Keisuke Nagao, Junko Misawa, Tetsuya Honda

**Affiliations:** ^1^ Department of Dermatology Hamamatsu Medical Center Hamamatsu Shizuoka Japan; ^2^ Department of Dermatology Hamamatsu University School of Medicine Hamamatsu Shizuoka Japan; ^3^ Dermatology Branch National Institute of Arthritis and Musculoskeletal and Skin Diseases National Institutes of Health Bethesda Maryland USA; ^4^ Department of Pathology Hamamatsu Medical Center Hamamatsu Shizuoka Japan; ^5^ Department of Rheumatology Hamamatsu Medical Center Hamamatsu Shizuoka Japan

## Abstract

Epstein‐Barr virus‐positive mucocutaneous ulcer (EBVMCU) is a B‐cell proliferative disorder that has been designated as a provisional entity in the 2017 World Health Organization classification for lymphoid neoplasms. While EBVMCU may contain varying numbers of cells with Hodgkin and Reed‐Sternberg cells‐like morphology, the clinical course is benign and must be distinguished from lymphomas. Patients who develop EBVMCU are commonly immunocompromised, with methotrexate (MTX) as the leading cause. Most previously reported cases of EBVMCU describe mucosal ulcers with very little documentation on skin lesions and its course. Here, we report a case of MTX‐associated EBVMCU of the lower leg that underwent spontaneous regression after MTX withdrawal, during which negative conversion of local Epstein‐Barr virus activation was confirmed.

## Graphical abstract

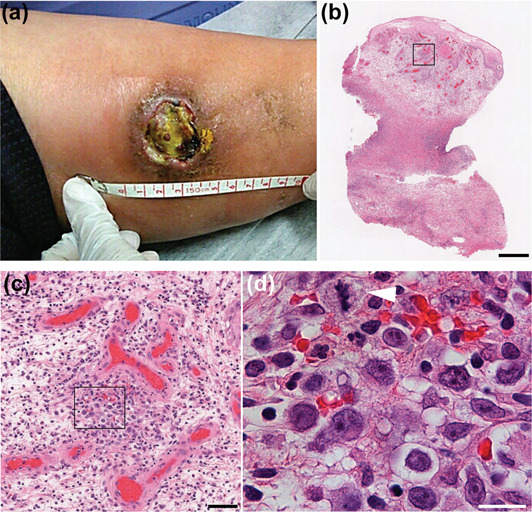

1

**What is already known about this topic?**

- EBVMCU is a rare B‐cell lymphoproliferative disease mostly affecting mucosal surfaces associated with immunosuppression. Clinical course is benign and should be distinguished from lymphoma.

**What does this study add?**

- We describe an even rarer case of cutaneous EBVMCU, which underwent complete regression with negative conversion of local EBV activation upon MTX withdrawal.

## 2 INTRODUCTION

Methotrexate (MTX) is an immunosuppressive drug that is commonly utilised for various autoimmune diseases such as rheumatoid arthritis (RA). However, MTX is associated with increased risk for lymphoproliferative disorders or lymphomas.^1^ Epstein‐Barr virus‐positive mucocutaneous ulcer (EBVMCU) was first reported in 2010^2^ and was designated as a provisional entity in the 2017 World Health Organization classification for lymphoid neoplasms^3^ with MTX as one of the leading cause.^4^ Currently, EBVMCU is considered as a spectrum of B‐cell lymphoproliferative diseases. Epstein‐Barr virus‐positive mucocutaneous ulcer commonly manifests as sharply circumscribed mucocutaneous ulcers.^2^ Notably, most cases occur on mucosal surfaces with only 10% of reported cases (10/100) manifesting in the skin.^4^ Epstein‐Barr virus‐positive mucocutaneous ulcer lesions contain varying numbers of cells with Hodgkin and Reed‐Sternberg cells‐like morphology, but the clinical course of EBVMCU is benign and must be distinguished from lymphomas.^2^ Alleviation of immunosuppression can lead to spontaneous regression of EBVMCU^4^ but how this course correlates with local or systemic EBV activation has not been described. Herein, we report a case of MTX‐associated EBVMCU of the lower leg that underwent spontaneous regression after MTX withdrawal, during which negative conversion of local but not systemic EBV activation was confirmed.

## 3 REPORT

**FIGURE 1:**
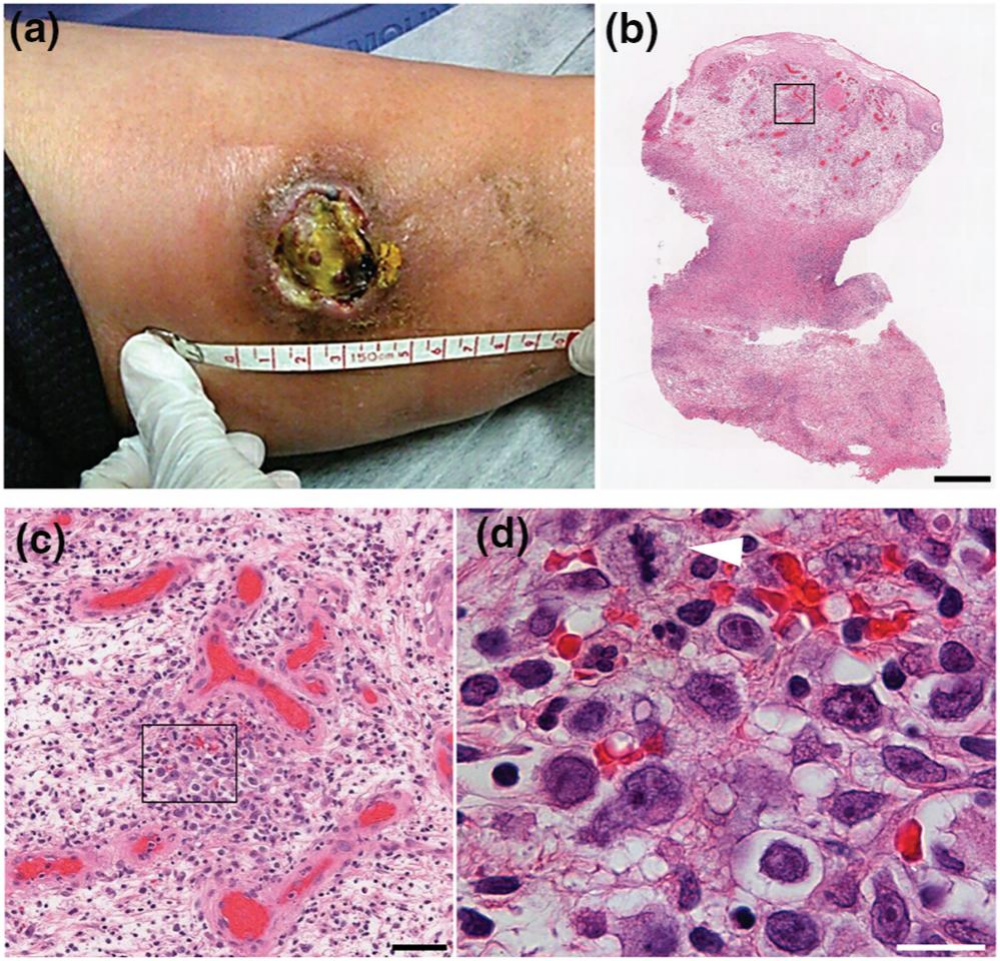
Clinical presentation and histological findings. (a) Clinical presentation of the left lower leg at administration. (b) Low, (c) mid and (d) high magnification of H&E staining. Rectangles in (b) and (c) depict the magnified areas in (c) and (d), respectively. Arrowhead in (d) depicts a mitotic cell. Scale bar = 500 μm in (b), 50 μm in (c), and 10 μm in (d)

A 74‐year‐old Japanese woman with a 30‐year history of RA had been treated with MTX at 8 mg/week for the past 5 years. Six months prior to her visit, she developed a red papule on her left lower leg that was possibly due to an insect bite, which gradually enlarged and progressed into a sharply circumscribed ulcer with necrotic surface (Figure 1a). The patient had high fever with redness and swelling around the ulcer, consistent with a secondary bacterial infection, for which antibiotic treatment was initiated. No active joint symptoms or superficial lymphadenopathies were appreciated. Differential diagnoses included vasculitis, infections, and neoplasia. Laboratory tests were notable for elevated Creactive protein (7.49 mg/dl) and rheumatoid factor (96 IU/ml). Ziehl‐Neelsen staining and *Mycobacterium avium* polymerase chain reaction on the tissue were negative. Chest and abdominal CT scans were unremarkable.

**FIGURE 2:**
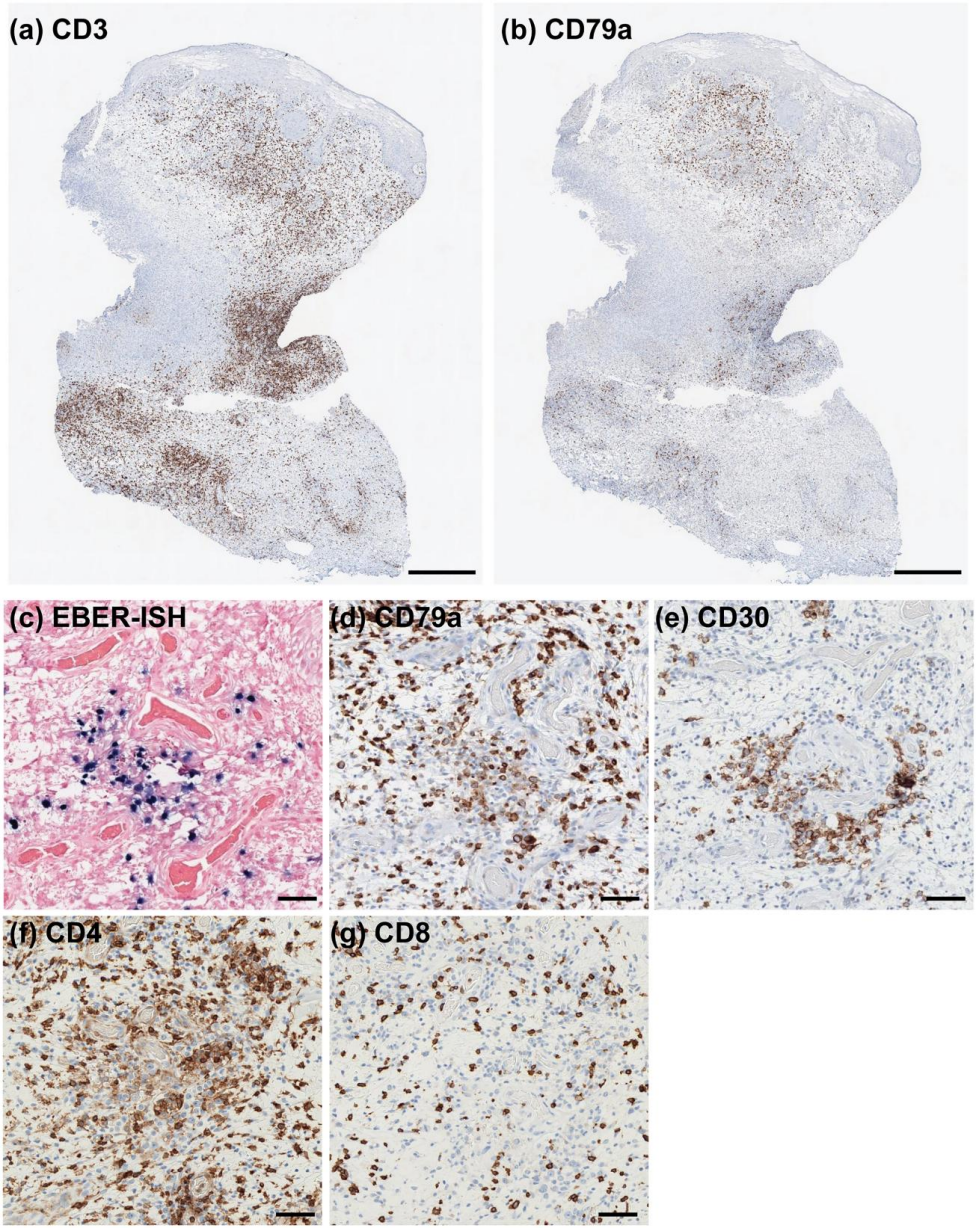
Low magnification of immunohistochemistry for (a) CD3 and (b) CD79a. (c) EBV‐encoded small RNA in situ hybridiszation (EBER‐ISH) showed positive staining in the nuclei of lymphocytic cells. Immunohistochemistry for (d) CD79a and (e) CD30 each highlight positive cells that accumulate in the EBER‐ISH^+^ areas. Immunohistochemistry for (f) CD4 and (g) CD8 highlights the predominance of CD4 T cells. Scale bar = 500 μm in (a and b), 50 μm in (c–g)

Punch biopsy from the ulcer revealed pseudocarcinomatous hyperplasia and hyper‐vascularity with dense lymphocytic infiltrate throughout the dermis and subcutis, some of which had large, atypical nuclei and occasional mitoses (Figure 1b–d). Immunohistochemistry revealed a mixed CD3^+^ T and CD79a^+^ B cell infiltrate with discrete islets of T cells lining the ulcer bottom (Figure 2a,b).

**FIGURE 3:**
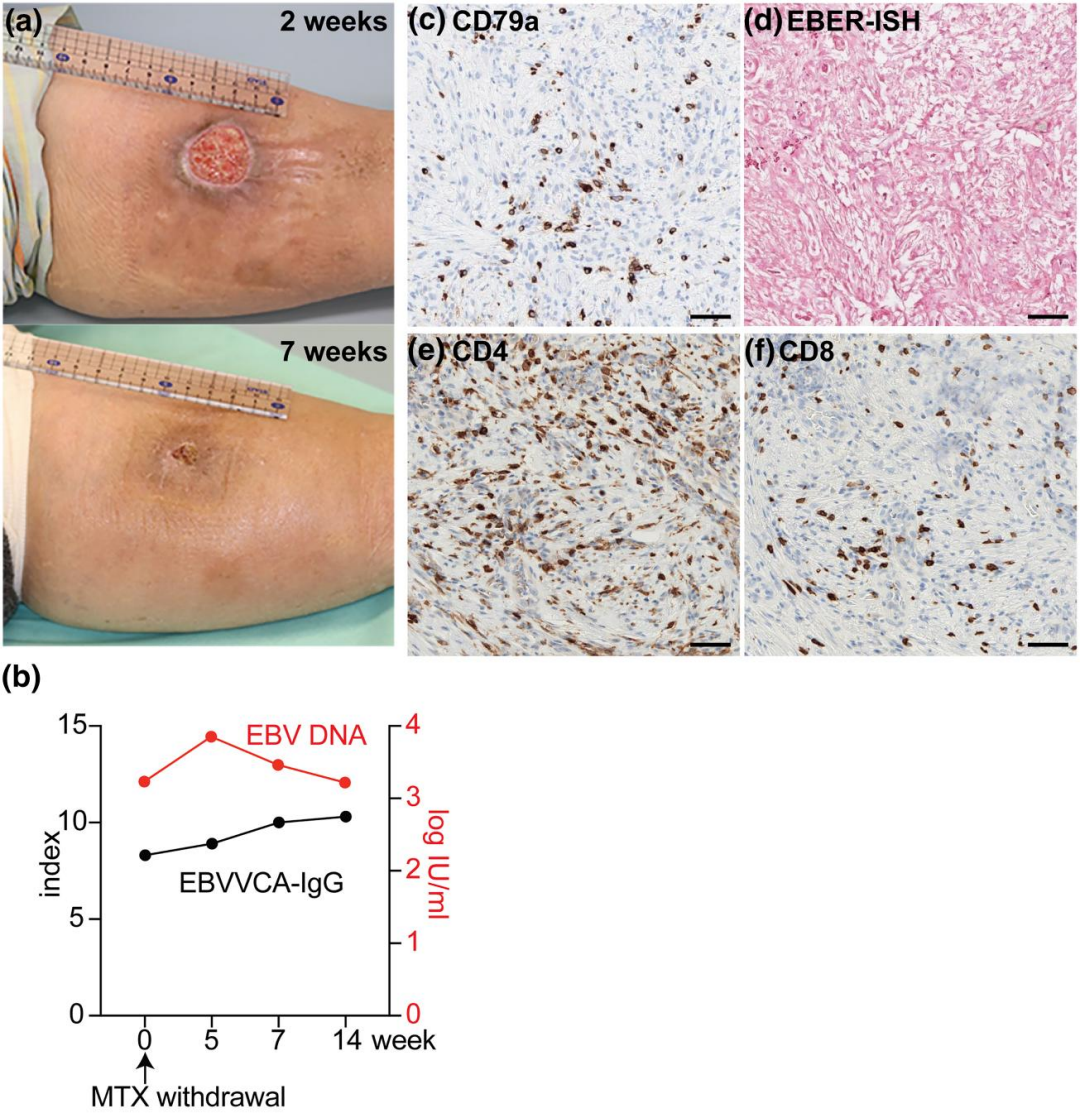
(a) Clinical presentation of Epstein‐Barr virus‐positive mucocutaneous ulcer (EBVMCU) two (upper panel) and 7 weeks (lower panel) post‐MTX withdrawal. (b) EBVVCA‐IgG levels (index, positive >1.0) and Epstein‐Barr virus EBV DNA levels (log IU/ml) at indicated timepoints. (c) Immunohistochemistry for CD79a, (d) EBV‐encoded small RNA in situ hybridiszation (EBER‐ISH), (e) CD4, and (f) CD8 from the re‐biopsy specimen at 5 weeks post‐MTX withdrawal. Scale bar = 50 μm in (b–e)

Given the non‐healing, sharply demarcated ulcer, lymphocyte‐rich histology with some atypism, and history of MTX use, EBVMCU was suspected. Thus, we performed EBV‐encoded small RNA in situ hybridisation (EBER‐ISH), which revealed positivity in the dermis and subcutis. EBER‐ISH pattern correlated with that of CD79a (Figure 2c,d), suggesting that EBV was active in a subset of mature B cells.^5^ EBER‐ISH^+^ area was also enriched with CD30^+^ cells (Figure 2e), a pathognomonic feature in EBVMCU.^2^ T cell infiltrates consisted of both CD4 and CD8 T cells, with predominance of the former (Figure 2f,g). Taken together, the patient was diagnosed with MTX‐associated EBVMCU. IgG against the EBV‐viral capsid antigen and EBV nuclear antigen were detected at 8.3 and 3.5 (positive >1.0 index), respectively. Epstein‐Barr virus DNA was detected in the blood (3.24 log IU/mL), indicating systemic activation. Methotrexate was discontinued considering EBVMCU and low RA activity, which led to rapid regression and healing of the ulcer at 7 weeks‐post MTX withdrawal (Figure 3a). IgG against the EBV viral capsid antigen slightly increased post‐MTX withdrawal, but EBV DNA levels remained detected until 14 weeks‐post MTX withdrawal, indicating persistent systemic EBV activation (Figure 3b). In contrast, skin re‐biopsy taken at 5 weeks‐post MTX withdrawal demonstrated that despite the presence of CD79a^+^ B cells, EBER‐ISH had converted negative (Figure 3c,d). To examine if MTX withdrawal led to increase T cell responses locally, we stained for CD4 and CD8 T cells, which revealed unchanged T cell constituents (Figure 3e,f). The patient remains free of EBVMCU at 4 months‐post MTX withdrawal and has not experienced exacerbation of RA.

## 4 DISCUSSION

Herein, we reported a case of EBVMCU of the left lower leg in an RA patient that was treated with MTX. Interestingly, blood analysis and re‐biopsy of the EBVMCU lesion post‐MTX withdrawal demonstrated that while systemic EBV reactivation persisted, discontinuation of MTX was sufficient to reverse local EBV reactivation and to induce regression of the skin ulcer. These data suggested that systemic EBV activation alone did not trigger EBVMCU but that it required MTX and perhaps other local exogenous cues, such as minor trauma or insect bites, which may lead to the infiltration and proliferation of EBV‐infected B cells and further to the formation of EBVMCU. Additionally, while it is well‐established that CD8 T cells confer protective immunity against EBV,^6^ we did not observe increased CD8 T cell infiltration post‐MTX withdrawal. Although the numbers of infiltrating T cells do not necessarily reflect their antigen‐specificity, functional or activation statuses, our results suggested that local reactivation of EBV in EBVMCU is not simply a result of MTX‐mediated T cell immunosuppression. It is possible that aberrant immunity due to autoimmunity, MTX‐induced immunomodulation, and exogenous cues all represent contributing factors that trigger pathology in EBVMCU.

In summary, MTX withdrawal was sufficient to induce spontaneous regression of EBVMCU that was accompanied by negative conversion of local EBV activation. These observations highlighted the benign nature of this condition distinct from that of lymphomas and reiterated the involvement of EBV in EBVMCU pathogenesis. This case also emphasises that the diagnosis of EBVMCU must be taken into consideration when immunocompromised patients present with non‐healing skin ulcers, which can commonly be mistaken as inflammatory or infectious lesions. Performing skin biopsies to confirm lymphocyte‐rich histology and local EBV activation in such cases would facilitate diagnosis, allowing care providers to assess if cessation of contributing agents is feasible in individual cases.

## CONFLICT OF INTEREST

The authors declare no conflict of interest.

## AUTHOR CONTRIBUTIONS

**K. Sakamoto:** Conceptualization; formal analysis; investigation; methodology; writing – original draft; writing – review & editing. **T. Baba:** Conceptualization; formal analysis; investigation; methodology; writing – review & editing. **H. Takatori:** Conceptualization; writing – review & editing; **K. Nagao:** conceptualization; funding acquisition; supervision; writing – review & editing. **J. Misawa:** Supervision; writing – review & editing. **T. Honda:** Supervision; writing – review & editing.

## Data Availability

The data that support the findings of this study are available from the corresponding author upon reasonable request.
